# Cognitive Failure in Adults with Spinal Cord Injury: A Valuable Adjunct Measure for Enhancing Cognitive Assessment and Rehabilitation Outcomes

**DOI:** 10.3390/neurolint15040087

**Published:** 2023-11-08

**Authors:** Ilaria Pozzato, Mohit Arora, Candice McBain, Nirupama Wijesuriya, Yvonne Tran, James W. Middleton, Ashley R. Craig

**Affiliations:** 1 John Walsh Centre for Rehabilitation Research, Northern Sydney Local Health District, St. Leonards, NSW 2065, Australiajames.middleton@sydney.edu.au (J.W.M.); a.craig@sydney.edu.au (A.R.C.); 2 Kolling Institute, Faculty of Medicine and Health, The University of Sydney, Sydney, NSW 2065, Australia; 3 George Institute for Global Health, Newtown, NSW 2042, Australia; nirupama.wijesuriya@gmail.com; 4 Australian Institute of Health Innovation, Macquarie University, North Ryde, NSW 2109, Australia; yvonne.tran@mq.edu.au

**Keywords:** spinal cord injury, cognitive impairment, cognitive failure, assessment, psychosocial, rehabilitation

## Abstract

Cognitive impairment is common in persons with spinal cord injury (SCI), impacting their daily functioning and rehabilitation. This study assesses the extent of self-reported cognitive failures in everyday life in persons with SCI and its relationships with objective neurocognitive measures and psychosocial factors, including depressive mood, anxiety, perceived control, and fatigue. The differences between forty-one adults with a chronic SCI and forty-one able-bodied controls were examined. The participants completed the Cognitive Failures Questionnaire (CFQ) to assess cognitive failure and neurocognitive tests assessing attention and executive functions, as well as a psychosocial assessment. The SCI group reported higher cognitive failure rates than the able-bodied group (31.7% versus 19%, *p* > 0.05). Objective neurocognitive tests did not significantly correlate with the CFQ scores in either group. However, the CFQ scores were positively associated with most psychosocial factors, even after controlling for covariates. The CFQ scores were significantly associated with depressive mood in persons with SCI. These findings highlight the importance of incorporating self-reported cognitive measures into neurocognitive assessments and rehabilitation planning for adults with SCI. Self-reports capture everyday cognitive challenges that objective tests may miss. Additionally, this study highlights the strong connections between cognitive failures and psychosocial issues, particularly mood disorders, emphasizing the need for comprehensive rehabilitation and psychosocial support post-SCI, addressing both cognitive and emotional wellbeing.

## 1. Introduction

Spinal cord injury (SCI) is a severe chronic condition involving a loss of motor and sensory function and associated secondary health problems, such as spasticity, cardiovascular and autonomic dysfunction, skin complications, psychological issues, pain, and cognitive impairment (CI), that require intense rehabilitation [1,2,3,4]. CI is prevalent following SCI, with estimates of up to 60% [5,6,7]. CI after SCI can be attributed to various factors, including co-morbid traumatic brain injury (TBI), autonomic dysregulation, neuroinflammation, fatigue, depression, polypharmacy, older age, or substance abuse [5,6,7,8,9,10].

Clearly, it is clinically important to assess cognitive function following SCI in order to maximize rehabilitation outcomes. However, there is an emerging recognition that several issues might undermine the accuracy and validity of assessing CI following SCI [11]. These issues include (i) methodological weaknesses, such as estimates of CI relying on cross-sectional evaluations; (ii) administrative weaknesses, including an absence of a gold standard definition for assessing CI; and (iii) the lack of neurocognitive screening tools specific to SCI that have demonstrated structural validity [11].

A further concern involves reliance on objective standardized neurocognitive tests alone for the assessment of CI. This approach, while valuable for specific diagnostic and research purposes, has been criticized for its limited practical relevance to “real-world” applications [12,13]. The critique centres on the idea that such tests, which are often conducted in controlled environments, may not always effectively capture the full range of an individual’s cognitive abilities or their capacity to apply these abilities to the complex, multifaceted challenges of daily life [14,15]. 

This limitation becomes particularly evident in the context of rehabilitation, where the focus is on enhancing a person’s social participation and overall wellbeing, extending beyond cognitive metrics to encompass the broader spectrum of an individual’s life, including their relationships, their engagement in meaningful activities, and their overall quality of life [16]. The International Classification of Functioning, Disability, and Health (ICF), established by the World Health Organization, serves as the gold standard for evaluating human functioning, disability, and health, especially within the context of rehabilitation. According to the ICF framework, an individual’s participation and overall wellbeing are closely tied to their cognitive, mental, and physical abilities, as well as the influence of various contextual factors [16]. 

Within this context, rehabilitation researchers have argued that a more comprehensive and relevant approach to the objectives of rehabilitation is needed to assess cognitive functioning, where broader dimensions of the ICF framework must be considered. One significant facet of this approach is the inclusion of self-reported cognitive failures as a complementary means of evaluation [12,13]. Self-reported cognitive failures offer a valuable perspective by directly tapping into people’s lived experiences of cognitive challenges and shortcomings. Arguably, this approach recognizes the intricate interplay between cognitive abilities, social participation, and contextual factors, offering a more nuanced and realistic portrayal of the cognitive challenges people experience in their life, rehabilitation, and overall journey towards an improved quality of life. A notable tool in this regard is the Cognitive Failures Questionnaire (CFQ) [12,17]. What makes the CFQ especially relevant is its multidimensional nature [18], capturing various dimensions of cognitive functioning and aligning with the ICF framework [16]. 

Previous research has linked a range of trait- and state-like factors to increased risks of cognitive failures [12,17]. For example, moderate relationships have been found between cognitive failures and psychological factors, such as low mood and elevated anxiety. However, the relationship between cognitive failures and objective neurocognitive data remains unclear, as research has only found small correlations between these two measures [12] and no research has been conducted on SCI populations. Furthermore, researchers have concluded that cognitive failure assessment is a potentially valuable addition to standard cognitive assessment, since it provides a broader dimension of cognitive functioning [12,13] by including self-reported cognitive ability and its impact on everyday functioning. 

In light of these findings, this paper presents research on adults with chronic SCI and comparisons with able-bodied controls. The main purpose was to examine the extent of self-reported cognitive failures and the relationships between cognitive failures and objective neurocognitive tests in the context of SCI. Additionally, the paper aims to investigate the connections between cognitive failures and common psychosocial problems, such as depressive mood, anxiety, low perceived control, and fatigue. It was hypothesized that (i) adults with SCI would have a higher rate of cognitive failures compared to able-bodied controls, (ii) no significant relationships would be found between cognitive failures and neurocognitive tests, (iii) cognitive failures, as indexed by CFQ scores, would be influenced by psychosocial factors like depressive mood and fatigue, and (iv) the interaction between cognitive failures (CFQ scores) and psychosocial factors is expected to exhibit variations between persons with SCI and able-bodied controls. 

## 2. Materials and Methods

### 2.1. Participants

In total, 41 adults with a chronic SCI and 41 able-bodied controls were included. Participants with SCI were recruited from specialist outpatient services, through advertising in relevant newsletters or via referrals from health professionals/acquaintances. The inclusion criteria for the SCI sample were: (i) a diagnosis of chronic SCI (>12 months post-injury), (ii) age between 18 and 70 years, (iii) proficiency in English, and (iv) living in the community. Able-bodied controls were recruited through convenience sampling, including approaching acquaintances and placing posters strategically in public areas. Controls were only admitted into the study if they were aged from 18 to 70 years, were proficient in English, and reported the absence of chronic physical or psychiatric diseases at the time of assessment. The 41 controls were chosen based on their similarities to the SCI group in terms of sex, age (±5 years), and education level. The selection was performed on a case-by-case basis from a pool of approximately 100 able-bodied participants who, along with the participants with SCI, had been enrolled into a study investigating the neuro-psychophysiological aspects of fatigue [10]. The level and extent (i.e., completeness) of the SCI were assessed by a medical specialist based on the International Standards for Neurological Classification of SCI (http://ais.emsci.org/). A total of 56% sustained an SCI in a motor vehicle crash and 22% from a fall. The majority (78%) of the SCI sample did not have a comorbid TBI. 

The study received ethical approval (ref id: 08 HAWKE/157/158) and the participants were entered into the study after obtaining their informed consent. Ethical standards were adhered to as required by the responsible human research ethics committee and the Helsinki Declaration, as revised in 2013.

### 2.2. Assessment Protocol

The study involved an experimental design with pre- and post-measurements of psychosocial function alongside a neurocognitive task. However, the current analysis focuses on a cross-sectional component. The assessment protocol has been reported in detail previously [10]. The participants were asked to refrain from consuming alcohol for 12 h prior to testing, and to refrain from consuming caffeinated food/beverages and smoking cigarettes two hours prior to the laboratory assessment. Both groups were assessed prior to participating in an extended task involving a one-hour regimen of validated neurocognitive tests presented online. The pre-task psychosocial assessment required around 40 min to complete, while the post-task psychosocial assessment took less than 10 min. The SCI and able-bodied participants completed the neurocognitive tasks by either pushing a button pad or clicking a mouse (an adapted chin button was used for people with high-level SCI). Pre-task psychosocial measurements were considered for this analysis. 

### 2.3. Cognitive and Psychosocial Measures

Cognitive failures were evaluated using the Cognitive Failures Questionnaire (CFQ) [17]. The Cognitive Failures Questionnaire (CFQ) is a valuable tool due to its multidimensional nature, which surpasses one-dimensional cognitive assessments by comprehensively evaluating a range of cognitive aspects that contribute to an individual’s cognitive functioning. This multifaceted approach enables a more nuanced understanding of how cognitive impairments manifest in daily life. What adds to its significance is its alignment with the ICF framework [18], a globally recognized standard for assessing human functioning and disability [16]. The CFQ’s correspondence with the ICF framework allows for a holistic evaluation that integrates cognitive failures with the broader spectrum of an individual’s functioning, considering cognitive, mental, and physical abilities, as well as contextual factors. This alignment promotes a comprehensive approach to understanding how cognitive challenges impact an individual’s daily life, participation, and overall wellbeing, thus facilitating tailored interventions and support strategies.

The CFQ is a widely recognized instrument designed to capture the frequency of lapses in cognitive control that disrupt planned thoughts and actions in everyday scenarios [17]. These lapses encompass a range of cognitive functions that people routinely experience in their daily lives, including perception, memory, and motor function [17]. The CFQ is a well-established tool known for its test–retest reliability, and has demonstrated predictive and criterion validity, making it a suitable choice for assessing cognitive failures in this study [19]. Furthermore, the questionnaire exhibits a strong internal reliability, ensuring that it consistently measures what it intends to. It is worth noting that the mean CFQ score for adults typically hovers around 33, with a standard deviation of 9 [20], providing a benchmark for average cognitive failure experiences. In this analysis, a cutoff score of 42, representing the CFQ mean plus one standard deviation (33 + 9 = 42), was employed, serving as a reference point for identifying individuals with a more elevated frequency of cognitive failures. This approach allows for a standardized evaluation of cognitive failures, enabling a deeper exploration of their impact and associations within the studied population. 

In the evaluation of cognitive function, specific neurocognitive tests were administered to assess distinct aspects of cognitive ability. The neurocognitive measures employed in this study were selected to serve as a brief yet effective cognitive assessment battery for evaluating attention and executive functioning, as the most prevalent impaired cognitive domains observed in persons with SCI according to a recent meta-analysis [5]. Our chosen assessments encompassed established and validated tools, specifically the digit span forward (attention domain) [21,22,23], reverse tests (executive domain) [24], and the Stroop test (attention and executive domains) [24,25,26], all of which were adapted from widely recognized standard cognitive assessment protocols. For the digit span, the participants were presented with a random sequence of digits, and asked to repeat them in either the order presented (forward span) or in reverse order (reverse span). Forward span specifically assesses (auditory) attention and short-term memory retention, while backwards span assesses (auditory) working memory and executive function [27]. The scoring involved tallying the total number of correct trials in both the forward and reverse digit span tasks. On the other hand, the Stroop test was employed to assess timed inhibition and information processing efficiency, particularly executive functions. In this test, the participants were tasked with selecting the correct colour of a word while ignoring the actual word itself, all within a time limit of 30 s. A higher score on the Stroop test signifies a greater processing speed and, by extension, superior executive functioning. [28].

Fatigue was assessed using the Chalder Fatigue Scale (CFS) [29]. The CFS is an 11-item self-report questionnaire of fatigue symptoms using four-point Likert items, in which “better than usual” scores 0, “no more than usual” 1, “worse than usual” 2, and “much worse than usual” scores 3, giving a range of 0–33. The CFS scale has an acceptable reliability and validity [29,30]. 

Depressive mood and anxiety were assessed using the 7-item Depression subscale (0 to 21) and the 7-item Anxiety subscale (0 to 21) of the Depression Anxiety Stress Scales 21 (DASS-21), a self-report questionnaire assessing anxiety, depressive mood, and stress [31,32]. High scores indicate elevated mood states. The DASS-21 has an acceptable reliability and discriminate validity [32]. 

Perceived control was assessed using the Lifestyle Appraisal Questionnaire Part 2 (LAQ2) [33]. It assesses perceived ability to influence life challenges. High scores indicate low perceived control. The LAQ2 has an acceptable test–retest reliability and internal reliability (Cronbach’s coefficient alpha of 0.89), as well as an acceptable construct validity and low social desirability [33]. 

The Medical Outcomes Study 36-Item Short-Form Health Survey (SF-36) is considered to be a reliable measure of quality of life (QoL) in SCI [34] and has an acceptable reliability and validity [35]. It assesses 8 domains; however, only 2 of interest are presented in this paper, that is, Role limit Physical and Bodily Pain. The SF-36 questionnaire was scored by summing and transforming raw data for each of the eight domains as per the formula in the SF-36 manual [36]. Higher scores on the eight domains suggest a higher QoL.

### 2.4. Analysis

Descriptive statistics were produced for the study variables. Pearson correlation analyses were conducted to determine the relationships between the CFQ scores and other variables. To explore how the CFQ scores were influenced by psychosocial factors like depressive mood, anxiety, perceived control, and fatigue, while accounting for the effects of covariates, four analyses of covariance (ANCOVA) were conducted. The CFQ scores were the dependent variable (DV) and the psychosocial factors were the independent variables (IV), and the analysis involved comparing these variables between two groups: persons with SCI and able-bodied controls (main group effect). The IVs were dichotomised into low versus high sub-groups based on mean values or cut-off values holding clinical significance while ensuring that the subgroup numbers remained reasonable. A group (persons with SCI, able-bodied controls) x IV (depressive mood, anxiety, perceived control, and fatigue) interaction effect was also studied to explore the differences in the interplay between the CFQ scores and psychosocial factors between the two groups. Covariates were selected based on theoretical considerations about factors that could influence cognitive impairment and thus impact the CFQ scores [7]. The covariates entered into the ANCOVA included age, sex, years of education, and, depending on the IV investigated, depressive mood, anxiety, fatigue, perceived control, SF-36 Physical-role, and SF-36 Bodily pain. The a priori statistical power to find valid differences using ANCOVA was calculated to be 90%. This level of statistical power indicates the likelihood of detecting valid differences in the study. The power calculation was based on several key factors, including the sample size (N = 82), the significance level (α = 0.05), the number of covariates (which was more than 5 in this study), and the effect size, which was determined to be 0.33. This effect size of 0.33 suggests that the study was designed to detect moderately sized effects [37]. All analyses were performed using Statistica Version 13 (https://www.statistica.com, accessed on 19 April 2023).

## 3. Results

Table 1 shows the socio-demographics, injury characteristics, and psychosocial factors for the SCI and able-bodied groups. The average age in the SCI group was 47.1 years (SD = 12), with a range from 21 to 70, and it consisted of 39 males and 2 females. Likewise, the control group had a mean age of 46.8 years (SD = 12) within the same age range and had 39 males and 2 females. Among the SCI participants, 27 had paraplegia and 14 had tetraplegia, with 23 individuals assessed by a medical specialist as having incomplete lesions and the remaining 18 having complete lesions. The mean time since injury was 16.5 years (SD = 14). Compared to the able-bodied group, the SCI group had significantly higher depressive mood, anxiety, and fatigue, as well as lower perceived control and years of education. As reflected by the CFQ mean score, there was a non-significant trend for the SCI group to have more mistakes in memory, perception, and motor function. Using the able-bodied CFQ mean + 1SD cut-off score of 42, which also corresponds to published norms [18], the percentage of participants with SCI who scored highly on the CFQ was 31.7% versus 19% in the able-bodied (*p* < 0.05). 

Table 2 shows the correlation analyses. The correlation analysis reveals interesting insights into the relationships between cognitive failures (CFQ scores) and various socio-demographic, injury, and psychosocial factors for the SCI and able-bodied control groups. For both groups, there were no significant associations found between the CFQ scores and the three neurocognitive test scores, while the CFQ scores were positively associated with most psychosocial factors. Notable findings include the strong positive correlations between the CFQ scores and factors like time since injury, DASS depressive mood, DASS anxiety, CFS total score, and LAQ 2 Perceived control in the SCI group. These results suggest that individuals with SCI who had higher scores on these psychosocial measures tended to report more cognitive failures. In contrast, the able-bodied group showed some correlations, but they were generally weaker, implying that the impact of these factors on cognitive failures was less pronounced in this group. The negative correlations with SF-36 Physical Role and SF-36 Pain in both groups suggest that those with better physical health tended to report fewer cognitive failures. 

**Figure 1:**
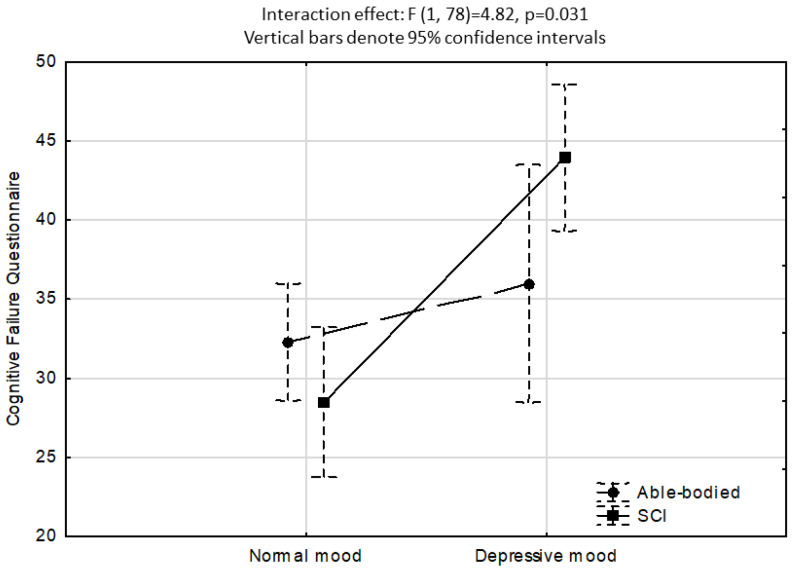
ANCOVA showing a significant interaction effect for CFQ scores as a function of normal versus elevated depressive mood. Covariates included sex, age, years educated, DASS anxiety, Chalder total fatigue, LAQ2 perceived control, SF-36 Physical role, and SF-36 Bodily pain.

None of the four ANCOVA analyses (IV: depressive mood, anxiety, perceived control, and fatigue) showed a significant main group effect (persons with SCI, able-bodied controls) on the CFQ scores (*p* > 0.05). In contrast, the ANCOVA investigating depressive mood as IV, as presented in Figure 1, showed that the effect for DASS Depressive mood was highly significant (normal mood < 5, high depressive mood ≥ 5; F1,78 = 12.8, *p* < 0.001), and so the group x depressive mood interaction for CFQ scores as a function of normal versus high depressive mood (F1,78 = 4.82, *p* < 0.05). While no differences in CFQ scores were found between the SCI and able-bodied groups for normal mood (Bonferroni post hoc test *p* > 0.05), the SCI high depressive mood sub-group had significantly higher CFQ scores than all the other sub-groups (Bonferroni post hoc *p* < 0.001). There were no significant CFQ differences between the able-bodied normal versus high depressive mood sub-groups. The ANCOVA investigating anxiety as IV showed that the effect for DASS anxiety was significant (low anxiety < 5, high anxiety ≥ 5; F1,78 = 12.9, *p* < 0.001) and that the SCI sub-group with elevated anxiety had a mean CFQ score 6 points higher than the able-bodied group mean. However, a non-significant group-anxiety interaction effect was found (F1,78 = 1.4, *p* > 0.05).

**Figure 2:**
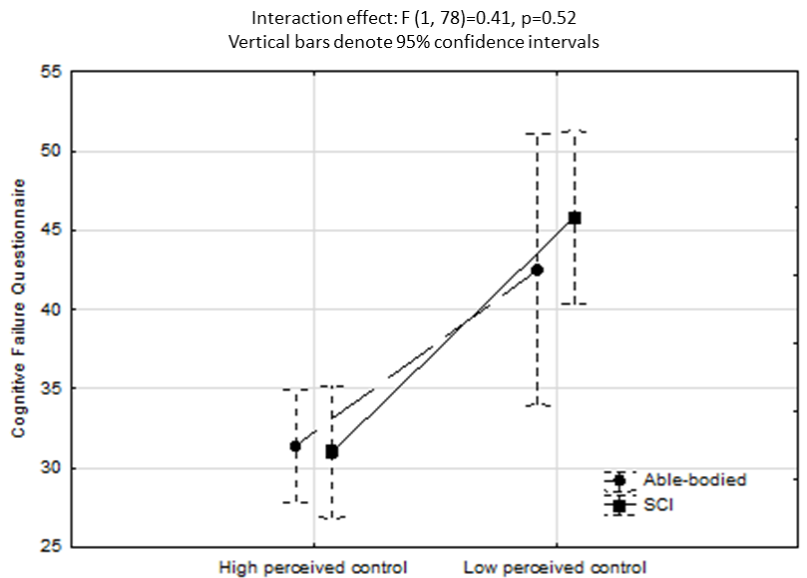
ANCOVA showing a non-significant interaction effect for CFQ scores as a function of perceived control. Covariates included sex, age, years educated, DASS depressive mood and anxiety, Chalder total fatigue, SF-36 Physical role, and SF-36 Bodily pain.

**Figure 3:**
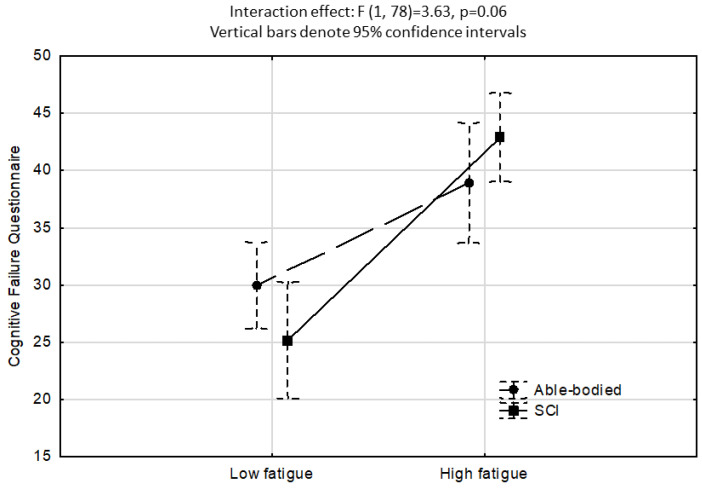
ANCOVA showing a non-significant interaction effect for CFQ scores as a function of low versus high fatigue. Covariates included sex, age, years educated, DASS depressive mood and anxiety, LAQ2 perceived control, SF-36 Physical role, and SF-36 Bodily pain.

Figure 2 shows the ANCOVA results investigating LAQ2 perceived control as IV. A significant effect was found for LAQ2 perceived control (high perceived control < 20, low perceived control ≥ 20; F1,78 = 20.1, *p* < 0.001), however, the interaction between groups and perceived control on CFQ scores was non-significant (F1,78 = 0.41, *p* > 0.05). Figure 3 shows the ANCOVA results investigating fatigue as IV, where a significant effect for fatigue (low fatigue < 12, high fatigue ≥ 12; F1,78 = 33.9, *p* < 0.001) but a non-significant group–fatigue interaction effect (F1,78 = 3.6, *p* = 0.06) were found.

## 4. Discussion

This study investigated self-reported cognitive failures in everyday life as an indicator of “real-life” cognitive ability in persons with SCI. It found low associations between cognitive failures and objective neurocognitive tests investigating attention and executive function domains, but strong associations with psychosocial factors such as elevated depressive mood. The findings suggest cognitive failures add value to neurocognitive evaluations and support holistic rehabilitation approaches in SCI populations. 

The finding of a higher rate of cognitive failures in the SCI group compared to the controls (over 30% of those with SCI compared to 19% of able-bodied participants) was not unexpected given the physical and neural impact of SCI, the high prevalence of associated secondary conditions (e.g., fatigue, autonomic dysfunction, and inflammation), and the influence of common treatments, such as polypharmacy. Given the findings of prior research, it was also no surprise that the SCI group scored lower in attention, inhibition, and information processing, as reflected by the forward digit and Stroop scores [5]. However, there was no statistically significant difference found in the reverse digit span test. This finding requires further investigation, as previous research has indicated that persons with SCI generally exhibit a lower performance in executive function and working memory compared to able-bodied individuals [5]. 

The lack of any significant correlation between either of the three neurocognitive tests (forward and reverse digit span and Stroop tests) evaluating attention and executive functioning—the most prevalent cognitive impairments in people with SCI [5]—with self-reported cognitive failures suggests that the CFQ measures a cognitive construct distinct from traditional neurocognitive measurements [12,18]. While this lack of association has been problematic for some researchers [12], others have highlighted the benefits of a self-reported measure of cognitive function reflecting a person’s perception of cognitive mistakes in everyday life [12,38], which has also been found to be associated with underlying neural capacity [39]. For example, it has been noted that people feel their cognitive functioning frequently varies with context, such as the time of day or week, and is subject to environmental and emotional influences (e.g., distractions, mood, and anxiety), with some days being perceived as less or more focussed and efficient [12,13]. Our results suggest that this is the case for adults with SCI, where, arguably, changes in personal and environmental contexts play a major role in individuals’ cognitive functioning [16], such as in an acute hospital setting versus living in the community. We therefore contend that a self-reported measure of cognitive function complements standard neurocognitive tests to capture day-to-day changes in cognitive performance and provide useful information on the cognitive deficits (“failures”) that most interfere with a person’s overall functioning and quality of life. 

Consistent with previous research, the SCI sample had significantly elevated levels of psychological morbidity. When the association between cognitive failures and high versus low depressive mood was calculated using ANCOVA, the results revealed a significant interaction effect between SCI versus able-bodied participants and depressive mood on cognitive failures. That is, participants with SCI and elevated depressive mood reported significantly more cognitive failures compared to the able-bodied and SCI sub-group with normal mood (see Figure 1). This is an important finding, as prior research has found a high occurrence of depression in adults with SCI, and those with SCI and cognitive impairment also have an increased risk of elevated depressive mood, especially after transitioning into the community [40]. In contrast, a weaker relationship was found between depression and cognitive function in the acute stages of an SCI [40,41], suggesting that early cognitive intervention may help to prevent subsequent mental health morbidity. 

While the interaction effects for cognitive failures as a function of perceived control (Figure 2) and fatigue (Figure 3) were not significant, the results indicate that poor perceived control and elevated fatigue increase vulnerability to making mistakes/errors in memory, language, and motor function, regardless of the presence of an SCI. Prior research has demonstrated that poor perceived control (or low self-efficacy) and elevated levels of fatigue are highly disabling for adults with SCI in terms of their quality of life and functionality [34]. Our findings reveal that poor perceived control and elevated fatigue were equally problematic for the group of healthy able-bodied individuals. While the directionality of this association cannot be established by this study, it suggests that perceived control, energy levels, and cognitive functioning are strongly interconnected aspects of human functioning, and this concurs with latest neurocognitive theories [42].

Considering the strong associations identified between cognitive performance and mental health problems within this sample, these findings provide practical guidance for improved management strategies and integrated psychosocial care [43]. This perspective aligns with the recommendations outlined in recently published guidelines [44] (https://aci.health.nsw.gov.au/__data/assets/pdf_file/0019/155233/ACI-Guide-health-professionals-psychosocial-care-adults-sci.pdf, accessed on 25 April 2023). For example, in addition to pharmacological and cognitive behavioural therapies for depression following SCI, rehabilitation efforts could consider cognitive retraining, speech-related social skills, and interventions aimed at enhancing vitality, such as regulated breathing and heart rate variability feedback [45]. Similarly, cognitive rehabilitation programs could be accompanied by psychosocial interventions that concentrate on improving mood regulation, self-efficacy, coping strategies, motivation, and adjustment following SCI [46,47]. 

### Study Limitations

The low to moderate sample size for each group (*n* = 41) limited the potential to show differences between the groups. For example, the post hoc power for the t-test for finding a CFQ difference between the groups was only 44%, with a small to moderate effect size [36]. However, the statistical power was higher at 65% when calculating the interaction effects in the ANCOVAs [36]. While the authors considered the brief cognitive battery employed in this study to be a foundational tool for delineating the prevalent cognitive deficits in persons with SCI, a more comprehensive cognitive assessment is recommended to extend the applicability of these findings regarding the associations between objective and self-reported cognition in people with SCI. Future research should increase sample size to at least 60 per group to increase the potential to reject the null hypothesis correctly.

## 5. Conclusions

The findings from this study highlight the importance of considering self-reported cognitive failures in the assessment of cognitive function and rehabilitation planning for adults with SCI. This measure captures everyday cognitive changes that may be missed by objective neurocognitive tests. Integrating individual perceptions of cognitive function and its impact on daily functioning adds meaningful value to neurocognitive evaluations within a comprehensive rehabilitation framework that addresses the physical, psychological, and social aspects of wellbeing for each person. Furthermore, the study highlights the strong association between cognitive failures and psychosocial issues, such as mood disorders, supporting the implementation of integrated rehabilitation approaches and psychosocial care for persons with SCI.

## Figures and Tables

**Table 1:** Socio-demographic, injury characteristics, and psychosocial factors for the two groups.

Variable	SCI Mean (SD)	Able-Bodied Mean (SD)
Male/Females, *n*	39/2	39/2
Age	47.1 (11.8)	46.8 (12.5)
Years educated	14.0 (2.6)	15.9 (2.0) *
Paraplegia, *n* (%)	27 (65.8)	-----
Time since injury	16.5 (14.0)	-----
DASS Depressive mood	9.5 (11.1)	2.9 (3.6) **
DASS Anxiety	5.3 (7.8)	2.7 (3.0) *
CFS total score	14.1 (9.0)	9.9 (6.6) *
LAQ2 Perceived control	19.8 (16.0)	14.0 (7.2) *
CFQ score	36.4 (14.0)	33.0 (9.4)
Forward digit span	5.4 (1.9)	6.4 (1.5) *
Reverse digit span	4.3 (2.0)	4.6 (2.0)
Stroop test (naming the colour)	8.5 (4.4)	10.7 (4.6) *

* *p* < 0.05 ** *p* < 0.01; SD: standard deviation; DASS: Depression Anxiety Stress Scale; CFS: Chalder Fatigue Scale; LAQ: Lifestyle Appraisal Questionnaire Part 2; and CFQ: Cognitive Failures Questionnaire.

**Table 2:** Pearson correlations between cognitive failures scores and socio-demographic, injury, and psychosocial factors for the SCI and able-bodied control groups.

Variable	SCI Group CFQ Score	Able-Bodied Group CFQ Score
Sex	0.01	0.01
Age	0.14	0.23
Years educated	0.10	0.12
Level of injury	0.26	----
Completeness of injury	−0.01	----
Time since injury	−0.51 **	----
DASS Depressive mood	0.53 **	0.20
DASS Anxiety	0.64 **	0.41 **
CFS total score	0.65 **	0.58 **
LAQ 2 Perceived control	0.63 **	0.47 **
SF-36 Physical Role	−0.33 *	−0.26
SF-36 Pain	−0.27	−0.39 *
Forward digit span	0.04	0.01
Reverse digit span	−0.08	0.08
Stroop test (naming the colour)	−0.03	0.09

* *p* < 0.05 ** *p* < 0.01; DASS: Depression Anxiety Stress Scale; CFS: Chalder Fatigue Scale; LAQ: Lifestyle Appraisal Questionnaire Part 2; CFQ: Cognitive Failures Questionnaire; and SCI: spinal cord injury.

## Data Availability

The data presented in this study are available on request from the corresponding author.
